# 经支气管镜支架植入联合化疗及阿帕替尼成功救治直肠癌支气管转移患者1例

**DOI:** 10.3779/j.issn.1009-3419.2017.09.12

**Published:** 2017-09-20

**Authors:** 秋琪 林, 玲 丁, 成 陈, 晔涵 朱

**Affiliations:** 215006 苏州，苏州大学附属第一医院呼吸与危重症医学科 Department of Respiratory Medicine, the First Affiliated Hospital of Soochow University, Suzhou 215006, China

**Keywords:** 直肠癌, 气管内转移, 气管支架, 阿帕替尼, Rectal cancer, Endobronchial metastasis, Bronchial stent, Apatinib

## Abstract

肺是肺外恶性肿瘤转移最常见的部位，而气道内转移少见。气道内转移最常见的肿瘤是肾癌、乳腺癌和结直肠癌。直肠癌晚期会出现肺转移，但气道内转移而无肺转移的少见，易误诊。我们报道1例直肠癌术后出现支气管内、胸膜腔、心包腔内转移，给予经支气管镜支架植入、化疗、靶向药物等综合治疗而获缓解的病例。诊治过程较为复杂，故有一定临床参考价值。

## Abstract (en)

The lungs are the most common sites of metastases from non-pulmonary malignancies. On the other hand, endobronchial metastases are rare. Various tumors have been associated with endobronchial metastasis, most commonly renal, breast and colorectal cancer. Advanced rectal cancerwith lung metastasis is common. However, endobronchial metastasis without lung metastasis of rectal cancer is rare, and easily misdiagnosed. We report one case of postoperative rectal cancer with endobronchial, pleuralcavity, pericardial cavitymetastasis, giving the comprehensive treatment of bronchial stentimplantation, chemotherapy, targeted drugs and remission. The process of diagnosis and treatmentis relatively complex, therefore it has a certain clinical reference value.

## 1 病例介绍

患者，女性，76岁，因“咳嗽、咳痰四月余，加重伴气喘半月”入住呼吸重症监护室（respiratory intensive care unit, RICU）。患者2015-06-28因咳嗽、咳痰到当地医院就诊，胸部计算机断层扫描（computed tomography, CT）（图 1）提示：左主支气管前见软组织影，左上叶支气管管腔狭窄伴阻塞性肺炎，左侧少量胸腔积液。患者拒绝行气管镜及胸腔穿刺等检查，仅予中医中药治疗。半月来患者咳嗽、咳痰症状加重，咳白粘痰，伴胸闷、气喘明显，夜间无法平卧，当地医院予抗感染、抗炎等治疗后症状不缓解。2015-11-13入院当天（第1天）查胸部CT（图 2）：与4月余前CT片比较，左主支气管前软组织影较前增大，左上叶支气管管腔已闭塞，伴有阻塞性肺炎，左侧胸腔积液较前增多，并出现少量心包积液及右侧胸腔积液。患者3年前曾行直肠癌手术，组织病理为腺癌，术后未行放疗及化疗。入院时呼吸频25次/分，不能平卧，口唇微绀，双肺可闻及广泛干、湿性罗音；心率100次/分，心律齐，心音低钝，未闻及心包摩擦音。入院后床边心超提示心包腔大量积液，紧急予心包穿刺及心包积液引流，患者气急症状明显缓解，每日心包积液引流量150 mL-250 mL，心包积液中癌胚抗原 > 1, 500.00 ng/mL；心包积液见癌细胞，倾向腺癌，患者血肿瘤全套中糖类抗原CA199为856.34 U/mL。结合患者肺部CT示左肺门占位伴左上叶支气管管腔闭塞，考虑为原发性肺腺癌（Ⅳ期）。入院3 d后心包腔注入香菇多糖治疗。循环肿瘤脱氧核糖核酸（circulating tumor deoxyribonucleic acid, ctDNA）检查结果示：表皮生长因子受体（epidermal growth factor receptor, *EGFR*）、间变性淋巴瘤受体酪氨酸激酶（anaplastic lymphoma receptor tyrosine kinase, *ALK*）、ROS原癌基因1，受体酪氨酸激酶（ROS proto-oncogene 1, receptor tyrosine kinase, *ROS-1*）、肝细胞生长因子受体（hepatocyte growth factor receptor, *HGFR*）、B-Raf原癌基因，丝氨酸/苏氨酸激酶（B-Raf proto-oncogene, serine/threonine kinase, *BRAF*）等基因无突变。患者为肿瘤晚期，病情危重，功能状态（performance status, PS）评分为3分，不宜行常规化疗，获患者及家属知情同意后，20 d后予培美曲塞800 mg d1单药抢救性治疗。患者胸闷气喘症状改善后出院。

**1:**
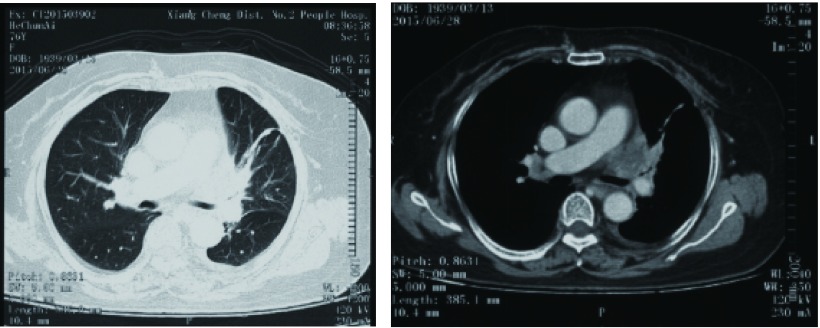
左主支气管前见软组织影，左上叶支气管管腔狭窄伴阻塞性肺炎，左侧少量胸腔积液（2015-06-28）。 Soft tissue shadow before left main bronchusis visible, left upper lobe bronchus luminal stenosis associated with obstructive pneumonia, a small amount of left pleural effusion (2015-06-28).

**2:**
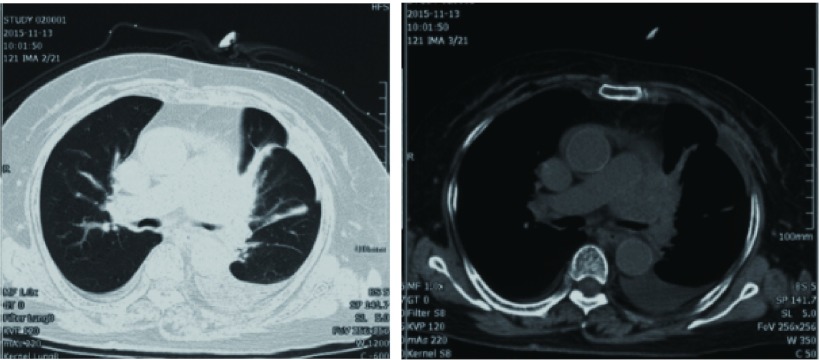
与4月余前CT片比较，左主支气管前软组织影较前增大，左上叶支气管管腔已闭塞，伴有阻塞性肺炎，左侧胸腔积液较前增多，并出现少量心包积液及右侧胸腔积液（2015-11-13）。 Compared with the CT of four months ago, soft tissue shadowbefore left main bronchus increase. Left upper lobe bronchial lumen is occlusive, accompanied with obstructive pneumonia. Left pleural effusion increase, and a small amount of pericardial effusion, and right pleural effusion (2015-11-13).

第35天后患者出现发热，热峰39.5 ℃，伴有畏寒及气急，第38天再次入住RICU，入院后胸部CT：左主支气管前占位病变，与2015-11-13胸部CT比较变化不明显，伴有阻塞性肺炎，左肺尚见少许斑片影，心包积液，左侧胸腔少量积液（图 3）。予抗感染等治疗后体温正常，又行第二次化疗（单药培美曲塞800 mg，d1）。因患者气急改善一直不明显，听诊肺部未闻及啰音，怀疑是否系心包积液导致胸闷气短可能，但心超检查未见明显心包积液；气急最终考虑系肿瘤引起的支气管阻塞导致阻塞性不张及阻塞性肺炎，于第60天患者终于同意接受行支气管镜检查，镜下见：右侧支气管管腔通畅；左上叶支气管管腔明显狭窄；左主支气管下端及近左上、下叶分嵴处均狭窄，局部粘膜高低不平，活检该部位粘膜送病理检查，予腔内测量；左下叶支气管通畅。3 d后在局麻及静脉麻醉下，行经支气管镜下气道支架植入术，经鼻进镜，见左主支气管下端外压性狭窄，经工作通道置入支架释放器，定位后释放支架（长3 cm、直径1.4 cm），并调整位置，辅以球囊扩张促进支架舒张。患者气管支架植入后胸闷气喘症状明显缓解。植入支架5 d后复查支气管镜，见左主支气管支架开口处脓痰附着，予以吸除，发现左上叶开口在支架下端开口范围之外，再予球囊扩张（分别予3个大气扩张2次，5个大气压扩张1次，每次扩张时间均为1 min），扩张后左上叶开口纳入支架下端开口范围内。病理诊断为：（支气管粘膜）见少量低分化腺癌浸润，免疫病理示癌细胞细胞角蛋白（cytokeratin, CK）、CK20、绒毛蛋白（Villin）、尾型同源核转录因子-2（caudal-related homeobox transcription factor 2, CDX2）阳性、Ki-67阳性（70%），CK7、甲状腺转录因子-1（thyroid transcription factor 1, TTF-1）、Napsin-A、P40、CK5/6均阴性，结合病史和免疫组化结果，符合直肠癌转移。最终确诊为直肠腺癌支气管转移，门诊于第70天开始予卡培他滨口服治疗5个疗程，停药后病情稳定。

**3:**
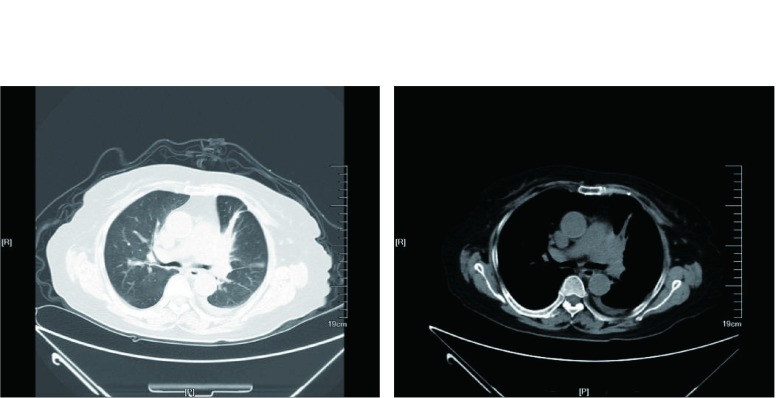
左主支气管前占位病变，与2015-11-13胸部CT比较变化不明显，伴有阻塞性肺炎（2015-12-23）。 Placeholder lesions before the left main bronchusis visible, no obvious changecompared with the chest CT of 2015-11-13, accompanied with obstructive pneumonia (2015-12-23).

第155天患者因胸闷气急加重至当地医院就诊，门诊输液治疗，未见好转，再次入住本院RICU。复查胸部CT：左主支气管支架植入术后改变，左肺上叶不张；纵隔淋巴结肿大，左侧胸腔积液；心包积液；右下肺结节，考虑转移（图 4）；与服用卡培他滨前（第41天）胸部CT（图 3）比较：左侧胸腔积液，心包积液明显增多。入院后予胸腔闭式引流、心包穿刺置管引流，患者胸闷气急症状改善，但咳嗽咳痰较前加重，双肺闻及湿性啰音，予抗感染治疗后咳嗽咳痰减轻。第175天起，给予血管内皮细胞生长因子受体2（vascular endothelial growth factor receptor 2, VEGFR-2）酪氨酸激酶抑制剂甲磺酸阿帕替尼，考虑患者一般情况欠佳，体能状态PS评分3分，无法耐受常规剂量，参考胃癌的治疗，我们给予该患者阿帕替尼500 mg *qd*治疗，后因为服药后出现指甲胀痛及全身不适，而改为阿帕替尼250 mg *bid*，患者咳嗽咳痰症状继续好转而出院。门诊随访至阿帕替尼服用7个月余，期间因为药物的副作用咽部疼痛及肛周疼痛而自行间断减量至500mg qod，患者病情基本稳定，生活能自理，颇为可惜的是，我们先后3次建议患者复查胸部CT，但患者自觉胸闷气急症状好转，拒绝复查。

**4:**
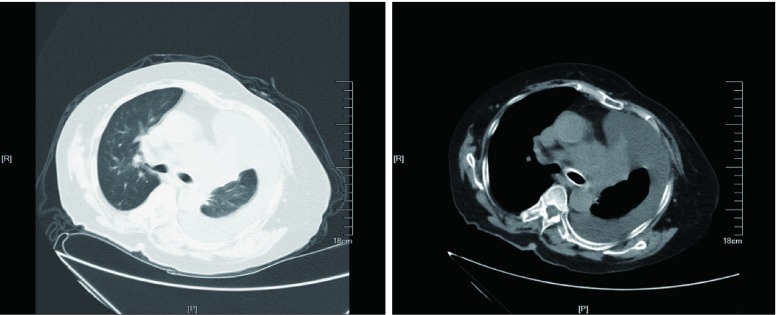
左支气管支架植入术后改变，左肺上叶不张，左侧胸腔积液（2016-04-25）。 Left bronchus change of bronchialstent implantation, left upper lobe atelectasis, left pleural effusion (2016-04-25).

## 2 讨论

尽管肺外恶性肿瘤发生肺转移很常见，但气道内转移却少见报道。因为支气管镜检查对于那些考虑肺转移的患者并非一项常规检查，这也许就是气道内转移诊断不足的原因之一^[1]^。Marchioni等^[2]^对174例患者进行的分析发现，肺外肿瘤发生气道内转移的比例占所有怀疑恶性肿瘤而进行气管镜活检病人的4%。较多恶性肿瘤可发生气道内转移，包括乳腺癌、结直肠癌、肾癌、卵巢癌、甲状腺癌、睾丸癌、鼻咽癌、前列腺癌、肾上腺癌、肝胰管壶腹腺癌等。其中以乳腺癌、结直肠癌和肾癌最为常见^[1, 3]^。结直肠癌患者发生气道内转移，常提示肿瘤已至晚期，因此对大多数患者来说，治疗目的在于缓解症状，而不是治愈。通常此时已不具备外科手术指征，患者若一般状况良好，应考虑化疗。气道内转移阻塞支气管的患者可受益于内镜介入手术，如内镜下的激光切除、冷冻治疗、支架植入和短距离放射治疗等，有时介入治疗还可结合放疗或化疗^[4, 5]^。

本例患者入院时胸闷气急明显，一般情况较差，因此治疗方面首先考虑缓解患者的症状：①心包腔、胸腔引流及抗感染治疗，改善心包填塞，改善肺通气换气功能；②心包积液见癌细胞，倾向腺癌，又有气道闭塞等特点，当时首先考虑原发性肺腺癌侵犯心包所致。培美曲赛是肺腺癌首选化疗药物之一，其副作用轻，且单药治疗有效。该患者PS评分差，我们予培美曲赛单药抢救性化疗后，患者症状改善，起到了救急的作用。③在患者气急症状没有明显缓解的情况下，临床考虑系阻塞性肺不张所致，在镜下得到证实后，植入支气管支架一枚，患者气急症状迅速改善。

气道内植入支架可立即扩张阻塞的支气管，缓解患者气急症状。气道内支架通常通过在DSA辅助下植入。本例是经支气管镜气道内支架植入术，该新技术有一定优势：①直视下可以观察支架到位情况及扩张情况；②可以随时调整支架高低位置；③支架扩张不良时可以马上发现，并辅以球囊扩张；④支架植入后若出现病情加重时，可以立即取出支架。我们植入支架后确实发现支架扩张不良，在支气管镜下予球囊扩张后得以释放，第3天复查发现仍有部分扩张不全时，再予球囊扩张后完全扩张，体现了经支气管镜气道内支架植入术的优势。

我们通过支气管镜术缓解了患者的气道症状，同时获取了气道粘膜病理。最后证实患者为直肠癌支气管转移。这给我们启示：直肠癌患者出现认为的第二肿瘤时，需要病理证实，以明确是第二肿瘤还是肿瘤转移，明确后可以获得对因治疗，改善预后。卡培他滨单药或与奥沙利铂联合是转移性结直肠癌的一线治疗方案之一，该患者在明确诊断后，即予卡培他滨单药口服，病情得到了一定的控制，生活质量明显改善。

服用卡培他滨5个疗程后患者病情进展，如何进一步选择治疗方案？我们想到了VEGFR-2酪氨酸激酶抑制剂甲磺酸阿帕替尼，它是全球首个在晚期胃癌被证实安全有效的小分子抗血管生成靶向药物，其在非小细胞肺癌、肝癌、结直肠癌中应用研究正在进行。在一项晚期恶性肿瘤患者的Ⅰ期研究中报道，有2例晚期直肠癌肺转移患者应用阿帕替尼后肺转移灶体积明显缩小，空洞形成及肿瘤密度减低，疗效评价为部分缓解（partial response, PR），这2例患者用药后至出现肿瘤进展时间分别为215天及255天^[6, 7]^。也有直肠癌肺转移使用联合化疗、西妥昔单抗联合化疗等2线-3线药物治疗后仍然进展，使用阿帕替尼取得短期初步疗效的个例报道^[8]^。因此，本例患者在停用希罗达后，接受了阿帕替尼500 mg *qd*的治疗，其咳嗽咳痰症状好转并出院。随访至阿帕替尼服用7个月余，期间在服药后第30天患者因咽部疼痛及肛周疼痛等药物副作用而自行减量至500 mg qod，患者病情基本稳定，生活能自理，达到临床缓解。在患者症状得到好转后，拒绝行胸部CT复查，因此我们无法判断是否达到影像学缓解。本例给我们提示，阿帕替尼可以试用于卡培他滨失效后的晚期直肠癌气道转移患者。
